# Probiotic Properties and Potentiality of *Lactiplantibacillus plantarum* Strains for the Biological Control of Chalkbrood Disease

**DOI:** 10.3390/jof7050379

**Published:** 2021-05-12

**Authors:** Massimo Iorizzo, Bruno Testa, Sonia Ganassi, Silvia Jane Lombardi, Mario Ianiro, Francesco Letizia, Mariantonietta Succi, Patrizio Tremonte, Franca Vergalito, Autilia Cozzolino, Elena Sorrentino, Sonia Petrarca, Antonio De Cristofaro, Raffaele Coppola

**Affiliations:** 1 Department of Agriculture, Environmental and Food Sciences, University of Molise, Via De Sanctis, 86100 Campobasso, Italy; bruno.testa@unimol.it (B.T.); silvia.lombardi@unimol.it (S.J.L.); m.ianiro@studenti.unimol.it (M.I.); f.letizia@studenti.unimol.it (F.L.); succi@unimol.it (M.S.); tremonte@unimol.it (P.T.); franca.vergalito@unimol.it (F.V.); autilia.cozzolino@unimol.it (A.C.); sorrentino@unimol.it (E.S.); decrist@unimol.it (A.D.C.); coppola@unimol.it (R.C.); 2 Conaproa, Consorzio Nazionale Produttori Apistici, 86100 Campobasso, Italy; sonia_petrarca@libero.it

**Keywords:** *Ascosphaera apis*, chalkbrood disease, *Lactiplantibacillus plantarum*, biocontrol, honeybee

## Abstract

*Ascosphaera apis* is an entomopathogenic fungus that affects honeybees. In stressful conditions, this fungus (due not only to its presence, but also to the combination of other biotic and abiotic stressors) can cause chalkbrood disease. In recent years, there has been increasing attention paid towards the use of lactic acid bacteria (LAB) in the honeybees’ diets to improve their health, productivity and ability to resist infections by pathogenic microorganisms. The screening of 22 strains of *Lactiplantibacillus plantarum*, isolated from the gastrointestinal tracts of honeybees and beebread, led to the selection of five strains possessing high antagonistic activity against *A. apis*. This study focused on the antifungal activity of these five strains against *A. apis* DSM 3116 and DSM 3117 using different matrices: cell lysate, broth culture, cell-free supernatant and cell pellet. In addition, some functional properties and the antioxidant activity of the five *L. plantarum* strains were evaluated. All five strains exhibited high antagonistic activity against *A. apis*, good surface cellular properties (extracellular polysaccharide (EPS) production and biofilm formation) and antioxidant activity. Although preliminary, these results are encouraging, and in future investigations, the effectiveness of these bacteria as probiotics in honeybee nutrition will be tested in vivo in the context of an eco-friendly strategy for the biological control of chalkbrood disease.

## 1. Introduction

The fungus *Ascosphaera apis*, belonging to the heterothallic Ascomycota phylum, is a major and widespread pathogen of honeybee (*Apis mellifera*) broods, causing chalkbrood disease and larval death [1]. This disease is economically important since it results in significant losses of both honeybees (under certain circumstances, it can kill colonies) and colony productivity [2], and indications suggest that its incidence may be increasing [3]. Recent research demonstrated that *A. apis* infection, together with other biotic and abiotic factors, induces oxidative stress and impairs the antioxidant defensive capacity of honeybee larvae [4].

Pathogenesis occurs when larvae ingest sexual spores of *A. apis* with their food. Inside the gut, the spores find the necessary anaerobic environment for their germination and extend into hyphal growth [5]. The infected larvae rapidly reduce their food consumption and then stop eating. The persistence of ascospores, which remain viable for many years on all surfaces inside the hive, provides a continuous source of infection [6]. Honeybees have several defense mechanisms to resist chalkbrood disease, including hygienic behavior [7]. However, if the potentially sporulating chalkbrood mummies are removed, hygienic behavior can increase rather than decrease transmission by exposing more individuals to the spores [8]. In addition, social insect species, such as *A. mellifera,* exhibit behaviors such as flower sharing to collect pollen and nectar, which might increase the transmission of persistent chalkbrood spores between colonies [9]. Drifting workers and drones may also contribute to the spread of infection [10].

Chalkbrood disease depends on several interacting aspects, such as the environment, the biological characteristics of both the host and the fungus (which may influence fungal pathogenesis and the transmission of the disease) and possible co-infections. Outbreaks may be increased by the disruption of the beneficial microbial community within a colony [11]. There is increasing knowledge on both the composition and the functions of the honeybee gut microbiota, which has led to the discovery of evidence of a link between balanced gut microbiota and honeybee health [12,13,14,15]. In particular, there is some evidence that *A. mellifera* gut microbiota may exhibit antifungal activity against *A. apis* [16,17,18]. A broad range of chemotherapeutic compounds have been tested to control chalkbrood disease over the years [19,20,21,22], but none have been able to control it properly. Furthermore, pesticides and antifungal chemicals have had serious impacts on the environment, honey quality and honeybee colonies themselves [23]. Therefore, there is great interest in developing alternative chalkbrood-controlling strategies.

In an interesting review, Gaggìa et al. [24] provided an overview of beneficial microorganism applications for the treatment of the main honeybee pathogens and their benefits in beekeeping production systems. Some more recent research has confirmed that the use of lactic acid bacteria (LAB) as probiotics could prevent certain diseases and improve honeybee health [25,26,27,28]. In particular, Tejerina et al. [29] recently demonstrated that the application of LAB (*Lactobacillus* *melliventris, Lactobacillus helsingborgensis* and *Lactobacillus kunkeei*) in sugar syrup over 5 months reduced larval mummification in chalkbrood disease by over 80%.

These data highlight that the administration of probiotic lactic bacteria in the honeybee diet can be a valid strategy for the biological control of chalkbrood disease. *Lactiplantibacillus plantarum* (formerly *Lactobacillus plantarum* [30]) is an important and ubiquitous LAB species characterized by extreme adaptability and genome plasticity. It has been isolated in many different environmental niches, such as fruit, vegetables, all types of fermented foods, meat and fish [31,32,33]. *L. plantarum* strains have also been isolated from different honeybee species [34,35,36,37]. Several authors have demonstrated that *L. plantarum* colonizes the adult *Drosophila melanogaster* gut and that it influences different aspects of the insect’s development and life, exerting a growth-promoting effect on larvae under nutrient scarcity [38,39,40,41,42]. Several authors have proved that *L. plantarum* has a broad capacity to inhibit the growth of different pathogens, and different strains exert inhibitory activity towards bacteria and fungi. In addition, chemically different compounds with antibacterial and antifungal activity have been characterized in culture filtrates [43,44,45,46], *L. plantarum* also exhibits antagonist activity against *Paenibacillus* *larvae*, the causative agent of the quarantine disease American foulbrood, which affects *A. mellifera* larvae and pupae [27,28,30,31,32,33,34,35,36,37,38,39,40,41,42,43,44,45,46,47]. Over the years, several studies have obtained relevant data supporting the probiotic properties of *L. plantarum* [48,49].

Suggested mechanisms by which probiotics may benefit the gut environment and the health of the host include improving intestinal barrier function through effects on the epithelium and mucus lining, producing antimicrobial substances, competing with pathogenic bacteria and antioxidative activity [50]. The ability of microorganisms to colonize is often considered one of the main selection criteria for potential probiotics, as their colonization is important for their activity. In addition, both their longer permanence in the mucosa of the host and their action as a biological barrier reduce or prevent pathogen colonization [51,52,53,54]. The ability of probiotic bacteria to adhere to intestinal epithelial cells involves extracellular polysaccharide (EPS) production and biofilm formation [51,52,53,54], and several *L. plantarum* strains are able to do both [48,49].

In this research, the antagonistic activity of five *L. plantarum* strains, isolated from the honeybee gut and beebread toward *A. apis* was assessed. The abilities of these lactic bacteria to produce EPSs and biofilms, as well as their antioxidant activity, were also evaluated. The final goal of this study was to evaluate the use of these *L. plantarum* strains as probiotics in the honeybee diet, and their potential use for the biocontrol of chalkbrood disease.

## 2. Materials and Methods

### 2.1. Microbial Cultures

In this study, 22 *L. plantarum* strains isolated from beebread, the midgut and the honey stomach of *A. mellifera* L. honeybees were used (Table S1). These bacteria belong to the Di.A.A.A. (Department of Agricultural, Environmental and Food Sciences) collection of the University of Molise [37]. As reference, *A. apis* DSM 3116 and *A. apis* DSM 3117 cultures (DSMZ: German Collection of Microorganisms and Cell Cultures GmbH) were used.

### 2.2. Screening for Antifungal Activity

The antifungal activity of the *L. plantarum* strains was assessed using the overlay method described by Magnusson et al. [55] with some modifications. The LAB strains were cultured in De Man, Rogosa and Sharpe (MRS) broth (Oxoid Ltd., Hampshire, UK) at 37 °C for 12 h. Then, they were inoculated with a central single streak of 2 cm on MRS agar plates, which were then incubated at 37 °C for 24 h under anaerobic conditions (GasPack anaerobic system, Sigma–Aldrich; St. Louis, MO, USA).

Fungal cultures from *A. apis* DSM 3116 and *A. apis* 3117 were cultured in Malt Extract Agar (MEA) medium (Oxoid Ltd., Hampshire, UK) under aerobic conditions at 28 °C for 5 days. Then, a 6 mm-diameter mycelial disc was removed, dissolved in physiological solution (0.9% NaCl) and vortexed for 5 min; 1 mL of the fungal suspension was then inoculated in a tube containing 10 mL of MEA soft agar (0.7% agar), which had been overlaid on the MRS agar plates previously inoculated with the LAB strains as described above. As a control, a plate containing MEA with the fungal suspension but without bacteria was used. After 72 h of incubation at 37 °C, the inhibitory activity of the *L. plantarum* strains was measured as the diameter (mm) of the clear zone around the bacterial streaks [56]. The tests were performed in triplicate.

### 2.3. Antifungal Activity Determination

#### 2.3.1. Spore Viability and Germination Test

Fungal cultures of *A. apis* DSM 3116 and *A. apis* 3117 were cultured in MEA medium at 28 °C for 15 days in aerobiosis. A spore suspension was obtained by washing the ascospores that formed on the surfaces of plates with 5–10 mL of 0.01% sterile Tween-80. The suspension was collected in a sterile 100 mL Erlenmeyer flask and loosened by shaking with sterile glass beads for 2 h. The germination test was conducted according to the procedure described by Jensen et al. [57] with some modifications. Briefly, sterile Teflon-coated slides (TEKDON, Myakka City, FL, USA) were placed in a sterile Petri dish lined with wet filter paper. Then, 100 μL of spore suspension (about 10^7^ spores/mL) was mixed with 400 μL of GLEN medium [57] and 100 μL of LAB culture (grown in MRS broth at 37 °C for 24 h), and 10 μL of this spore/GLEN/LAB (SGL) mixture was placed onto the Teflon-coated slides. A spore/GLEN (SG) mixture without LAB cultures was used as a control. To stimulate germination, the Petri dish was exposed for 10 min to 9–13% CO_2_ [58] using an AnaeroGen sachet in a 3.5 L jar (Oxoid; Basingstoke, UK), and after 32 h at 34 °C in aerobiosis, we counted the spores directly on the Teflon slide. About 100 spores were counted in three different fields of view on the slide using a phase contrast microscope at 400× magnification (Axioplan, Zeiss; Göttingen, Germany). Spores were considered germinated when the length of a hypha was longer than the length of the diameter of the spore. All the chemical compounds were supplied by Sigma–Aldrich (St. Louis, MO, USA). The tests were conducted in triplicate.

#### 2.3.2. Inhibition of Radial Mycelial Growth

The inhibitory activity against the *A. apis* 3116 and *A. apis* 3117 strains was determined according to Iorizzo et al. [59] using the following matrices of LAB cultures: broth culture (BC), cell-free supernatant (CFS), cell pellet (CP) and cell lysate (CL).

To obtain the matrices, each *L. plantarum* strain was cultivated in MRS broth and incubated at 37 °C for 12 h, reaching a cell concentration of 10^8^ CFU/mL. This culture, without any treatment, was the BC matrix. Then, 5 mL of this bacterial culture was centrifuged at 8000 rpm for 15 min at 4 °C; the resulting supernatant was sterilized by filtration (0.22 µm-pore-size cellulose acetate filter) to obtain the CFS matrix. For the CP matrix, the remaining pellet was washed and resuspended in 5 mL of physiological solution. To obtain the CL matrix, 5 mL of bacterial culture (BC) was centrifuged, and the pellet was washed, resuspended in 5 mL of physiological solution and then subjected to three cycles of sonication (Labsonic M; Sartorius, Germany) at 12 W for 30 s, with a 60 s pause between the cycles to promote cellular lysis [60].

For each matrix (BC, CP, CFS and CL), 5 mL was added to 15 mL of MEA; this preparation was then poured into 90 mm Petri dishes. After the solidification of the medium, a mycelial disc (6 mm in diameter) of each *A. apis* strain was placed in the middle of the Petri dish, which was then incubated at 37 °C under aerobiotic conditions. The antifungal activity was evaluated by measuring the hyphal radial growth (diameter) after 8 days of incubation and expressed as the percentage of inhibition using the following formula: % I = [1 − (Ds/Dc)] × 100, where Ds is the hyphal diameter of the sample and Dc is the hyphal diameter of the control (MEA with fungus only). The experiments were performed in triplicate.

### 2.4. Biofilm Production

Biofilm production was evaluated as described by Cozzolino et al. [61] with some modifications. The *L. plantarum* strains were grown overnight at 37 °C in MRS medium. The bacterial cells were harvested by centrifugation at 8000 rpm for 10 min at 4 °C, washed twice with phosphate-buffered saline (PBS) solution (Sigma–Aldrich, St. Louis, MO, USA), resuspended at 10^6^ CFU/mL in MRS broth without sugar and in MRS broth supplemented with 5%, 10% and 20% glucose, fructose or sucrose under aerobiotic and anaerobiotic conditions (GasPack anaerobic system, Sigma–Aldrich, St. Louis, MO, USA). Three 200 µL aliquots of each bacterial suspension were transferred to a 96-well polystyrene microtiter plate. Wells filled with uninoculated culture media were used as negative controls. The microtiter plates were incubated for 24 h at 37 °C. The medium was then removed from each well, and the plates were washed three times with a sterile physiological solution to remove unattached cells. The remaining attached cells were fixed with 200 µL of 99% methanol (Sigma–Aldrich, St. Louis, MO, USA) per well. After 15 min, the methanol was removed, and the cells were left to dry. Then, 200 µL of 2% Crystal Violet (Liofilchem; Roseto degli Abruzzi, Italy) was placed in the wells for 5 min. The excess stain was then removed by washing three times with sterile saline solution. After the plates were air-dried, the adherent cells were resuspended in 160 µL of 33% (*v*/*v*) glacial acetic acid (Sigma–Aldrich, St. Louis, MO, USA). The values of absorbance at 580 nm, measured using an automated Multilabel Counter (PerkinElmer 1420), represented the biofilm formation capacity. The experiments were performed in triplicate.

### 2.5. Exopolysaccharide (EPS) Assay

#### 2.5.1. Production and Isolation of EPSs

Microbial EPSs are not permanently attached to the microbial cell surface and exist in two forms depending on their location: cell-bound EPSs, which closely adhere to the bacterial surface (bound exopolysaccharides; EPS-b), and EPSs that are released into the surrounding medium (released exopolysaccharides; EPS-r).

For each bacterium, 200 mL of MRS medium was inoculated with 1% (*v/v*) overnight precultures grown in the same medium. After incubation at 37 °C for 48 h, the cultures (10^8^ CFU/mL) were centrifuged at 15,000× *g* for 15 min at 4 °C. The pellets were washed twice with sterile water and then centrifuged again at 15,000× *g* for 15 min at 4 °C and subjected to EPS-r and EPS-b extractions. The screening and extractions of EPS-r and EPS-b were carried out as described by Tallon et al. [62]. The final fractions were dried to constant weights. As a control, MRS broth without bacterial inoculum was used. The tests were conducted in triplicate. All the chemical compounds were supplied by Sigma–Aldrich (St. Louis, MO, USA).

#### 2.5.2. Antifungal Activity of EPSs

The fractions of EPS-b and EPS-r, obtained from 20 mL of MRS medium, were rehydrated with 5 mL of physiological solution and added to 15 mL of MEA for antifungal activity tests against *A. apis* 3116 and *A. apis* 3117 using the same technique described in Section 2.3. The corresponding fractions of non-inoculated MRS medium were used as controls. The tests were conducted in triplicate.

### 2.6. Antioxidant Activity

#### 2.6.1. Bacterial Culture Matrices and Cell Protein Assay

Overnight cultures (10^6^ CFU/mL) of the *L. plantarum* strains in LM medium (Table S2) were centrifuged at 13,000 rpm for 5 min at 4 °C, and the obtained supernatants (CFS_LM_) were used directly for the antioxidant activity assay.

Cell pellets (CPs) were divided into two aliquots to determine their protein content and antioxidant activity. For total cell protein extraction, the CP was resuspended in 1 mL of Tris-buffered saline (TRIS) solution at pH 7.5; 20 mM containing ethylenediaminetetraacetic acid (EDTA) 5 mM and MgCl_2_ 5 mM, and then subjected to three cycles of sonication at 12 W for 30 s, with a 60 s pause between the cycles, using a Labsonic M. The suspension was used for protein measurement according to Di Martino et al. [63] using a BioSpectrometer (Eppendorf, Hamburg, Germany). The total protein concentrations, expressed as µg/mL, were calculated by means of a calibration curve where bovine serum albumin (BSA) was used as a standard.

For antioxidant activity, the CP was washed twice with sterile water and resuspended in 200 µL of ethanol/water (40/60). The cell pellet suspensions were sonicated (12 W for 30 s, with a 60 s pause between the cycles) and, after 12 h of storage at −20 °C, centrifuged at 13,000 rpm for 15 min at 4 °C. The supernatants (CES) were used for the evaluation of antioxidant activity. All the reagents used in this experiment were from Sigma–Aldrich (St. Louis, MO, USA). All the experiments were performed in triplicate.

#### 2.6.2. Antioxidant Activity Assay

The total antioxidant activity (TAA) of the CFS_LM_ and CES, obtained as described above, was evaluated using the 2,2 azino-bis 3-ethylbenzothiazoline-6-sulfonic acid (ABTS·+) radical cation method according to Re et al. [64], with some modifications. Briefly, ABTS was dissolved in water to a concentration of 7 mM. ABTS radical cations (ABTS·+) were produced by reacting the ABTS stock solution with 2.45 mM potassium persulfate (final concentration) and allowing the mixture to stand in the dark at room temperature for 24 h before use. The ABTS·+ solution was diluted with citrate buffer (pH 4.0) to an optical density (OD) of 0.700 at 734 nm. Then, 100 µL of CFS_LM_ and CES were mixed with 900 µL of the ABTS·+ solution. The OD was measured at 734 nm after 4 min in the dark at room temperature using a BioSpectrometer (Eppendorf, Hamburg, Germany). Ascorbic acid was used as the standard for the calibration curve. The antioxidant activity of CFS_LM_ was expressed as µg ascorbic acid/mL; the antioxidant activity of CES was expressed as the ratio (*w/w*) between ascorbic acid (ng) and protein (µg; BSA equivalents). All the reagents used for this experiment were from Sigma–Aldrich (St. Louis, MO, USA). All the experiments were performed in triplicate.

### 2.7. Statistical Analysis

All the data obtained from three independent experiments are expressed as mean ± standard deviation (SD). Statistical analysis was performed using an analysis of variance (ANOVA) followed by Tukey’s multiple comparison test. Statistical significance was attributed to *p*-values < 0.05. SPSS software (IBM SPSS Statistics 21) was used for the analysis. The heatmap of biofilm production was generated using ClustVis web tool [65].

## 3. Results

### 3.1. Antifungal Activity

In a preliminary antifungal test, all 22 *L. plantarum* strains showed antifungal activity but with different intensities (Table S1).

The *L. plantarum* strains LP8, LP25, LP86, LP95 and LP100 caused inhibition zones more than 2 cm in diameter and were selected for subsequent analysis.

In the anti-germinative tests, no significant differences were observed between the control (SG) and the samples containing the cultures (SGL) of the *L. plantarum* strains (Table S3).

**Figure 1:**
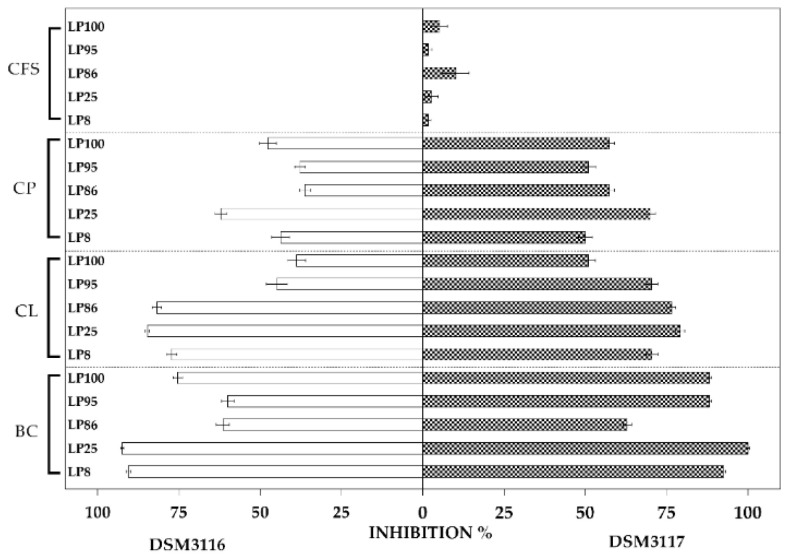
Inhibition (%) of *A. apis* DSM 3116 and *A. apis* DSM 3117 (radial growth) on Malt Extract Agar (MEA) plates after 8 days using culture broth (CB), cell pellet (CP), cell-free supernatant (CFS) and cell lysate (CL) from the *L. plantarum* strains.

The results of the inhibition of the radial mycelial growth of the *A. apis* 3116 and *A. apis* 3117 strains by the various matrices of *L. plantarum* cultures are summarized in Figure 1.

The results of the various tests show that there were significant differences between the radial growth percentage values obtained using different matrices. The numerical data are reported in Table S4.

After 8 days, the *L. plantarum* broth cultures (BCs) caused greater inhibition of the two fungi than the other matrices did, with values between 60.0% (LP95) and 92.4% (LP25) against *A. apis* 3116, and 62.9% (LP86) and 100% (LP25) against *A. apis* 3117.

**Figure 2:**
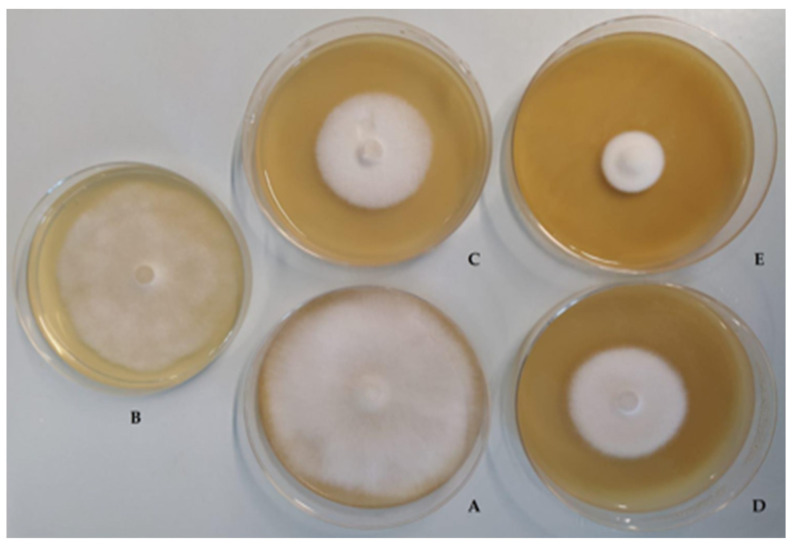
Inhibitory activity of *L. plantarum* LP100 against *A. apis* DSM 3116 after 8 days on MEA agar plates. A: *A. apis* (control); B: *A. apis* + CFS (cell-free supernatant); C: *A. apis* + CL (cell lysate); D: *A. apis* + CP (cell pellet); E: *A. apis* + BC (broth culture).

The cell lysates (CLs) inhibited the fungi more than the CP and CFS matrices. In particular, they caused inhibition rates between 38.8% (LP100) and 84.8% (LP25) for *A. apis* 3116, and between 50.9% (LP100) and 79.2% (LP25) for *A. apis* 3117. The cell pellets (CPs) showed inhibitory activity ranging from 36.3% (LP86) to 62.1% (LP25) against *A. apis* 3116, and from 50.0% (LP8) and 69.8% (LP25) against *A. apis* 3117. The cell-free supernatants (CFSs), overall, showed less inhibitory activity, which was found to be between 1.7% (LP8 and LP95) and 10.2% (LP86) for *A. apis* 3117, and they did not inhibit *A. apis* 3116. Figure 2 shows the inhibitory activity of *L. plantarum* LP25 against *A. apis* DSM 3117 after 8 days on MEA agar plates. EPS-b and EPS-r did not inhibit *A. apis* 3116 or *A. apis* 3117.

### 3.2. EPS and Biofilm Production

The amounts of EPS produced by the five *L. plantarum* strains are reported in Table 1. The EPS-r values, expressed as mg/mL, were obtained for each bacterial culture (10^8^ CFU/mL) in MRS broth. The EPS-b values are expressed as the ratio between EPS-b (μg) and cell protein (µg, BSA equivalents) obtained for each bacterial culture (10^8^ CFU/mL) in MRS broth. The data show statistically significant differences (*p* < 0.05). The EPS-r values were between 0.82 (LP95) and 1.56 mg/mL (LP25), while the EPS-b values were between 1.96 (LP95) and 8.82 mg/mL (LP8).

**Figure 3:**
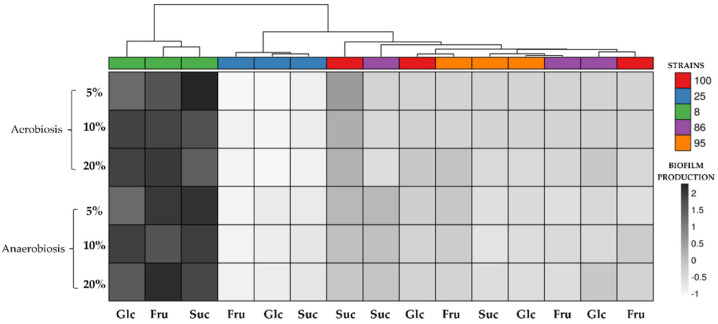
Biofilm production of *L. plantarum* LP8, LP25, LP86, LP95 and LP100 strains in aerobiotic and anaerobiotic conditions and with different sugar concentrations (5%, 10% and 20% (Glc: glucose; Fru: fructose; Suc: sucrose)). This figure was generated using ClustVis web tool [65] https://biit.cs.ut.ee/clustvis/ (accessed on 4 December 2020).

Figure 3 shows a heatmap in which the *L. plantarum* strains are clustered based on their different capacities to form biofilms in different media and environmental conditions. The biofilms were assessed by measuring the optical density (OD), and the numerical data are shown in Table S5. All the tested *L. plantarum* strains were able to produce biofilms in all the conditions, but to different degrees, depending on the concentration and type of the added sugar. *L. plantarum* LP8 produced, under all the conditions, greater amounts of biofilm than the other strains.

In all the tests, the anaerobiotic condition almost always favored the production of biofilms. In particular, the *L. plantarum* LP8 strain always produced more biofilm than the other strains under this condition. The anaerobiotic condition favored biofilm production in the tests with both glucose and sucrose for the LP25 strain, in the sucrose tests for the LP86 strain and in the fructose tests for the LP95 and LP100 strains.

All the bacterial strains tended to produce increasing amounts of biofilm as the sugar concentration increased, although there were often no significant differences (*p* > 0.05). Once again, the LP8 strain stood out because it produced increasing amounts of biofilm under the conditions of greater osmolarity of the sugar syrup; the differences for this strain were almost always significant (*p* < 0.05), except in the test conducted under aerobiosis with the addition of sucrose.

### 3.3. Antioxidant Activity

The antioxidant activities of the CFS_LM_ and CES matrices are shown in Table 1. The *L. plantarum* strains produced different results for each of the two matrices. The antioxidant activity values of the CFS_LM_, expressed as ng of ascorbic acid/mL, were between 20.01 (LP100) and 37.45 (LP8). Those of the CES, expressed as the ratio between ascorbic acid (μg) and cell protein (μg BSA equivalents), were between 0.11 (LP100) and 0.17 (LP8).

## 4. Discussion

### 4.1. Antifungal Activity

Our purpose was to investigate the ability of *L. plantarum* to inhibit two different *A. apis* strains, DSM 3116 and DSM 3117. The results suggest that sensitivity to the bacterial cultures may be species- and not strain-dependent. Future in vivo tests will be performed to verify the antifungal activity of *L. plantarum* against wild *A. apis* strains.

Our study demonstrated that the *L. plantarum* strains did not affect the germination capacity of fungal spores, while these LAB exhibited the ability to inhibit the vegetative form of *A. apis* in vitro. The mycelial hyphae of this fungus, which are responsible for its virulent action, penetrate the peritrophic membrane and gut wall barrier to enter the honeybee hemocoel. The pressure caused by the septate hyphae and the enzymatic activity favor access to the interstitial space between the muscle fibers of infected larvae [66,67]. The epithelial cells of the larval gastrointestinal tract are protected from pathogen colonization by several mechanisms exerted by commensal microbiota, including competition for adhesion sites or nutrient sources and producing antimicrobial substances [51,52,68,69].

Many other researchers have shown that the antimicrobial activity of LAB is primarily attributed to the CFS, in which several antimicrobial compounds are found, including organic acids (lactic, acetic, formic, propionic, butyric, hydroxylphenylactic and phenylactic acids) and other inhibitory substances (e.g., carbon dioxide, hydroperoxide, fatty acids and bacteriocins) [70,71,72,73,74,75,76,77,78,79,80,81,82].

Our tests of *A. apis* inhibition demonstrated that all five *L. plantarum* strains had strong antifungal activity. High inhibition occurred with the use of the broth cultures (BC), which was most likely due to an interaction between several factors. In addition, the inhibitory effects obtained using the cell pellet (CP) and cell lysate (CL) were stronger than those obtained with the cell-free supernatant (CFS). Our results suggest that there may be synergy between various compounds, extra- and intracellular, that substantially increases the overall antifungal activity. This has also been hypothesized by other researchers [51,52,53,54,55,56,59,60,61,62,63,64,68,69,70,71,72,73,74,75,76,77,78]. Our tests showed that the EPS-b and EPS-r fractions did not inhibit *A. apis* 3116 and *A. apis* 3117. This suggests that the higher inhibitory effect of the CL compared to the CFS was probably due to the release of antifungal compounds from the bacterial cytoplasm after cell lysis.

The mechanisms behind the inhibition may involve some individual compounds that can cause membrane destabilization (such as fatty acids or peptides), proton gradient interference (such as organic acids or peptides) or enzyme inhibition (such as hydroxy acids). In addition, there may be some synergistic and/or additive effects involving various compounds [83].

The antifungal compounds contained in the BC and CL matrices need to be investigated in future research, and after their identification and purification, we plan to use them in anti-germination tests on *A. apis* spores.

The antifungal properties of the *L. plantarum* strains shown in vitro do not axiomatically result in health benefits for honeybee colonies. It is therefore necessary to assess the role that these bacteria play in maintaining honeybee wellbeing and the contribution they can provide for the biological control of chalkbrood disease. In particular, we are testing the effects of sugar syrups enriched with lysates or live and active cultures of these *L. plantarum* strains, added to the diets of honeybee colonies in vivo/in situ.

### 4.2. EPS and Biofilm Production

Our results show that these five *L. plantarum* strains are able to produce EPSs and biofilms. As a result, these bacteria can persist in the intestine, where there is an abundant flow of sugars, enzymes and water and the constant invasion of foreign microbes following the ingestion of flower nectar during foraging [15,84,85,86,87,88,89,90,91]. The germination of *A. apis* spores occurs in the midgut lumens of infected honeybee larvae. The hyphae penetrate the peritrophic membrane and gut epithelium, and then invade larval tissues [3]. The inhibition of *A. apis* mycelial growth is an important key step for preventing the colonization of the intestinal cavity. Adhesion to the intestinal wall and the formation of biofilms by probiotic bacterial antagonists of pathogenic fungi could constitute an obstacle to the development and consequent invasive action of fungal mycelia.

Our tests also confirmed that EPS and biofilm production are strain-dependent, as documented by other researchers [92]. *L. plantarum* LP8 produced the largest quantities of EPS-b and biofilm, demonstrating that exopolysaccharides linked to the bacterial wall are important in the composition and architecture of biofilms [93,94,95].

The formation of EPSs and the development of a biofilm are also affected by other factors, including surface properties and environmental parameters [96,97,98]. Our results show that anaerobiotic conditions and increased osmolarity often significantly favor biofilm production (Table S5). This suggests that the microaerophilic/anaerobic conditions of the intestinal tract can favor the production of biofilms and the resulting intestinal colonization by these bacteria.

In the future, it will be necessary to perform this test with cell lines to confirm the adhesion of the five selected *L. plantarum* strains to the epithelial cells.

### 4.3. Antioxidant Activity

Oxidative stress is important in eukaryotic organisms and can cause severe negative effects. Reactive oxygen species (ROS) are the causative agents of oxidative stress, and they are produced during normal metabolic processes. Insects have a range of antioxidant enzymes, mainly composed of superoxide dismutase (SOD), catalase (CAT) and peroxidase (POD). Glutathione peroxidase (GPX) and glutathione reductase (GSR) can also remove ROS [99,100,101].

Detoxification enzymes play a critical, crucial role in honeybees exposed to biotic and abiotic stressors through ecological interactions with their environments (nutritional and thermal stress, parasites, heavy metals and/or pesticides) [102,103,104,105,106,107,108,109,110]. Oxidative stress has been reported to play an important pathological role in honeybee diseases. Even during the excessive proliferation of pathogens, the intestinal epithelium produces and releases high levels of ROS, causing significant oxidative stress [111,112,113]. Li et al. [4] recently reported that *A. apis* infection induced oxidative stress in honeybee larvae, and decreased levels of the metabolites involved in combating oxidative stress could compromise the antioxidant defenses of the infected larvae. The specific activities of antioxidant enzymes (CAT, GST and SOD) and the levels of metabolites (taurine, docosahexaenoic acid and l-carnitine) involved in combating oxidative stress were significantly decreased in the guts of infected honeybee larvae.

Increased attention has been paid over the last decade to the use of LAB as natural antioxidants. Some LAB strains have enzymatic and nonenzymatic antioxidant activity and promote the production of antioxidant enzymes, decreasing the risk of ROS accumulation during the ingestion of food, thereby reducing oxidative damage [114,115,116,117,118].

We assessed antioxidant activity using the ABTS assay, which is considered one of the most sensitive techniques [119] and a valid method for determining the antioxidant activity of both hydrophilic and lipophilic extracts [120]. All five *L. plantarum* strains showed antioxidant activity in the CFS_LM_ and CES matrices, and this suggests that their antioxidant activities may be due to different substances (e.g., intracellular antioxidant enzymes, nonenzymatic antioxidant components such as glutathione, cell surface proteins or polysaccharides, etc.), which need to be investigated in greater detail.

These bacteria, if used as probiotics in the diets of honeybees, could limit oxidative stress due to pathogenic *A. apis* fungi and other biotic and abiotic factors.

## 5. Conclusions

The *L. plantarum* strains used in our experiments have been shown to possess substances biologically active against *A. apis* fungi. These results confirm the potentially antagonistic role of *L*. *plantarum* against pathogenic microorganisms that use the digestive channels of honeybees as the sites of infection [47]. Moreover, our findings indicate the ability of the *L. plantarum* LP8, LP25, LP86, LP95 and LP100 strains to produce EPSs and form biofilms, which are prerequisites for potential candidates to be used as probiotics in the honeybee diet. In addition, the antioxidant properties of the tested bacterial strains can help to increase the tolerance of these insects to endogenous and exogenous oxidative stress. The obtained results encourage the design of strategies to improve honeybee health through nutritional approaches or the modulation of the gut microbiota using beneficial microbes and open up a new horizon for fighting honeybee pathogens.

Future research activities will involve the investigation of the nature of the antifungal compounds and evaluate the effects of these *L. plantarum* strains on honeybee health and productivity, and their efficacy in chalkbrood disease biocontrol in vivo/in situ.

## Figures and Tables

**Table 1:** EPS production in MRS medium after 48 h and antioxidant activity in LM medium of *L. plantarum* LP8, LP25, LP86, LP95 and LP100 strains. All values are expressed as mean ± standard deviation (n = 3). Different lowercase letters (a–d) in each row indicate significant differences (*p* < 0.05).

	*L. plantarum* Strains				
	LP8	LP25	LP86	LP95	LP100
CFS_LM_	37.45 ± 0.40 ^c^	36.88 ± 0.40 ^c^	25.73 ± 0.81 ^b^	22.30 ± 0.05 ^a^	20.01 ± 0.81 ^a^
antioxidant activity *					
CES *	0.17 ± 0.02 ^b^	0.16 ± 0.01 ^b^	0.12 ± 0.01 ^a^	0.14 ± 0.01 ^a^	0.11 ± 0.00 ^a^
antioxidant activity **					
EPS-r	1.40 ± 0.03 ^b^	1.56 ± 0.50 ^c^	1.28 ± 0.10 ^b^	0.82 ± 0.07 ^a^	1.49 ± 0.06 ^b^
EPS-b	8.82 ± 0.11 ^d^	2.23 ± 0.09 ^a^	5.05 ± 0.12 ^b^	1.96 ± 0.08 ^a^	5.54 ± 0.10 ^c^

* CFS_LM_ antioxidant activity expressed as ascorbic acid (ng)/mL; ** CES antioxidant activity expressed as ratio of ascorbic acid (μg)/cell protein (μg BSA equivalents); EPS-r values expressed as mg/mL; EPS-b values expressed as the ratio of EPS-b (μg)/cell protein (μg BSA equivalents).

## Data Availability

Not applicable.

## Supplementary Materials

The following are available online at https://www.mdpi.com/article/10.3390/jof7050379/s1. Table S1: List of 22 *L. plantarum* strains, Table S2: Letizia medium (LM) composition, Table S3: Spore germination, Table S4: Fungal inhibition, Table S5: Biofilm production.
