# Supplementary material for: Probiotic Properties and Potentiality of Lactiplantibacillus plantarum Strains for the Biological Control of Chalkbrood Disease
Source: J Fungi (Basel). 2021 May 12;7(5):379. doi: 10.3390/jof7050379 (PMC8151994; doi:10.3390/jof7050379)
Supplement: Supplementary file 1 [file jof-07-00379-s001.zip › jof-1155325-supplementary/Table S1.pdf]

Origin	Strains	*A. apis* **DSM 3116 (ZOI cm)**	*A. apis* **DSM 3117 (ZOI cm)**
Bee bread	LP5	0.80±0.06	0.70±0.07
	LP25	2.20±0.10	2.40±0.03
	LP36	0.60±0.06	0.60±0.03
	LP39	0.70±0.06	0.60±0.06
	LP 86	2.20±0.09	2.10±0.05
	LP88	0.80±0.04	0.90±0.07
	LP99	0.60±0.04	0.60±0.05
	LP100	2.30±0.08	2.50±0.09
Honey stomach	LP8	2.60±0.07	2.20±0.09
	LP22	0.90±0.08	1.00±0.10
	LP28	0.60±0.05	0.60±0.04
	LP30	0.70±0.06	0.70±0.02
	LP55	0.60±0.04	0.60±0.03
	LP59	0.60±0.05	0.60±0.06
	LP75	0.60±0.06	0.60±0.05
	LP81	0.80±0.07	1.00±0.05
	LP90	0.80±0.07	0.80±0.04
	LP91	0.70±0.05	1.00±0.01
	LP95	2.10±0.07	2.20±0.02
	LP109	0.60±0.04	0.80±0.06
Mid gut	LP7	0.60±0.07	0.60±0.04
	LP33	0.60±0.06	0.90±0.03

Table S1. List of 22 *L. plantarum* strains: origin and antifungal activity screening (zone inhibition diameter - ZOI cm), on to MEA agar plates, against *A. apis* DSM 3116 and *A. apis* DSM 3117. Results are shown as mean ± standard deviation (n=3).
