# Supplementary material for: Probiotic Properties and Potentiality of Lactiplantibacillus plantarum Strains for the Biological Control of Chalkbrood Disease
Source: J Fungi (Basel). 2021 May 12;7(5):379. doi: 10.3390/jof7050379 (PMC8151994; doi:10.3390/jof7050379)
Supplement: Supplementary file 1 [file jof-07-00379-s001.zip › jof-1155325-supplementary/Table S2.pdf]

COMPOUNDS	g/L
D-Glucose	5
D-Fructose	5
Potassium dihydrogenphosphate	2
Sodium acetate	5
Ammonium citrate tribasic	5
Magnesium sulfate	0.2
D-RIbose	1
D-Xylose	1
D-Maltose monohydrate	1
DL -Malic acid	1
L-Lysine monohydrate	0.1
L -Histidine	0.1
L-Tyrosine	0.1
L-Phenylalanine	0.1
L-Cysteine HCL monohydrate	0.1
Glycine	0.1
Thiamine Hydrochloride	1
Manganese (II) sulfate	0.05

Table S2. Letizia medium (LM) composition.
