# Supplementary material for: Probiotic Properties and Potentiality of Lactiplantibacillus plantarum Strains for the Biological Control of Chalkbrood Disease
Source: J Fungi (Basel). 2021 May 12;7(5):379. doi: 10.3390/jof7050379 (PMC8151994; doi:10.3390/jof7050379)
Supplement: Supplementary file 1 [file jof-07-00379-s001.zip › jof-1155325-supplementary/Table S3.pdf]

A.apis strains	Spore germination %					
	**SG**	**SGL-LP8**	**SGL-LP25**	**SGL-LP86**	**SGL-LP95**	**SGL-P100**
**DSM 3116**	84±3[a]	83±1[a]	80±3[a]	82±4[a]	78±5[a]	84±2[a]
**DSM 3117**	75±5[a]	70±3[a]	76±2[a]	75±6[a]	75±2[a]	78±6[a]

Table S3. Spore germination (%) in SG (spore/GLENN) and in SGL (spore/GLENN/LAB) mix. Results are shown as mean ± standard deviation (n=3). Different lowercase letters, in each row, indicate significant differences ($p < 0.05$).
