# Supplementary material for: Probiotic Properties and Potentiality of Lactiplantibacillus plantarum Strains for the Biological Control of Chalkbrood Disease
Source: J Fungi (Basel). 2021 May 12;7(5):379. doi: 10.3390/jof7050379 (PMC8151994; doi:10.3390/jof7050379)
Supplement: Supplementary file 1 [file jof-07-00379-s001.zip › jof-1155325-supplementary/Table S4.pdf]

A.apis strains	**Matrices**	**Inhibition %**				
		**LP8**	**LP25**	**LP86**	**LP95**	**LP100**
DSM 3116	BC	90.6±0.7^{Dc}	92.4±0.5^{Dc}	61.5±2^{Ca}	60.0±1.9^{Da}	75.3±1.5^{Db}
	CL	77.2±1.6^{Cc}	84.7±0.6^{Cd}	81.8±1.4^{Dd}	44.9±3.3^{Cb}	38.8±2.8^{Ba}
	CP	43.7±2.8^{Bb}	62.1±1.8^{Bc}	36.3±1.7^{Ba}	37.8±1.5^{Ba}	47.6±2.6^{Cb}
	CFS	< 1 ^{Aa}	< 1 ^{Aa}	< 1 ^{Aa}	< 1 ^{Aa}	< 1 ^{Aa}
DSM 3117	BC	92.5±0.7^{Dc}	100.0±0.0^{Dd}	62.9±1.3^{Da}	88.1±0.8^{Db}	88.2±0.6^{Db}
	CL	70.5±1.8^{Cb}	79.2±1.4^{Cc}	76.5±1.3^{Cc}	70.4±1.8^{Cb}	50.9±2.2^{Ba}
	CP	50.0±2.2^{Ba}	69.8±1.7^{Bc}	57.4±1.6^{Bb}	51.0±2.2^{Ba}	57.3±1.7^{Cb}
	CFS	1.7±0.8^{Aa}	2.7±2.1^{Aa}	10.2±4^{Ab}	1.7±1.2^{Aa}	5.1±2.4^{Aa}

Table S4. Inhibition (radial growth %) against *A. apis* DSM 3116 and *A. apis* DSM 3116 on to MEA agar plates, after 8 day in aerobiosis, using broth culture (BC), cell pellet (CP), cell free supernatant (CFS) and cell lysate (CL) of *L. plantarum* strains. Results are shown as mean ± standard deviation (n=3). Different uppercase letters (A-H), in each column, and different lowercase letters (a-d), in each row, indicate significant differences ($p < 0.05$).
