# Supplementary material for: Probiotic Properties and Potentiality of Lactiplantibacillus plantarum Strains for the Biological Control of Chalkbrood Disease
Source: J Fungi (Basel). 2021 May 12;7(5):379. doi: 10.3390/jof7050379 (PMC8151994; doi:10.3390/jof7050379)
Supplement: Supplementary file 1 [file jof-07-00379-s001.zip › jof-1155325-supplementary/Table S5.pdf]

| Biofilm production ($OD_{580}$) | | | | | | | |
|---|---|---|---|---|---|---|---|
| **Sugar** | **Concentration** | **Environmental condition** | **LP8** | **LP25** | **LP86** | **LP95** | **LP100** |
| Glucose | 5% | Aerobiosis | 0.507±0.011$^{Ac}$ | 0.103±0.008$^{Aa}$ | 0.220±0.021$^{Ab}$ | 0.227±0.014$^{Ab}$ | 0.219±0.016$^{Ab}$ |
| | 10% | | 0.709±0.021$^{Cc}$ | 0.108±0.005$^{Aa}$ | 0.242±0.016$^{Ab}$ | 0.230±0.019$^{Ab}$ | 0.241±0.019$^{Ab}$ |
| | 20% | | 0.778±0.010$^{Dd}$ | 0.100±0.006$^{Aa}$ | 0.298±0.020$^{Bc}$ | 0.223±0.020$^{Ab}$ | 0.297±0.014$^{Bc}$ |
| | 5% | Anaerobiosis | 0.609±0.012$^{Bd}$ | 0.154±0.008$^{Ba}$ | 0.227±0.013$^{Ab}$ | 0.207±0.019$^{Ab}$ | 0.265±0.022$^{Bc}$ |
| | 10% | | 0.782±0.047$^{Dc}$ | 0.150±0.011$^{Ba}$ | 0.232±0.011$^{Ab}$ | 0.224±0.016$^{Ab}$ | 0.263±0.013$^{Bb}$ |
| | 20% | | 0.810±0.032$^{De}$ | 0.152±0.008$^{Ba}$ | 0.346±0.024$^{Cd}$ | 0.238±0.019$^{Ab}$ | 0.294±0.007$^{Bc}$ |
| Fructose | 5% | Aerobiosis | 0.574±0.018$^{Ac}$ | 0.106±0.005$^{Aa}$ | 0.221±0.020$^{Ab}$ | 0.224±0.015$^{Ab}$ | 0.216±0.009$^{Ab}$ |
| | 10% | | 0.701±0.011$^{Bc}$ | 0.125±0.008$^{Aa}$ | 0.231±0.020$^{Ab}$ | 0.232±0.018$^{Ab}$ | 0.230±0.010$^{Ab}$ |
| | 20% | | 0.795±0.008$^{Cd}$ | 0.121±0.008$^{Aa}$ | 0.234±0.013$^{Ab}$ | 0.326±0.025$^{Bc}$ | 0.231±0.008$^{Ab}$ |
| | 5% | Anaerobiosis | 0.765±0.024$^{Cd}$ | 0.112±0.011$^{Aa}$ | 0.197±0.008$^{Ab}$ | 0.296±0.009$^{Bc}$ | 0.197±0.013$^{Ab}$ |
| | 10% | | 0.735±0.024$^{Bd}$ | 0.132±0.010$^{Aa}$ | 0.223±0.012$^{Ab}$ | 0.295±0.025$^{Bc}$ | 0.295±0.017$^{Bc}$ |
| | 20% | | 0.860±0.041$^{Dd}$ | 0.143±0.009$^{Aa}$ | 0.239±0.018$^{Bb}$ | 0.294±0.009$^{Bc}$ | 0.293±0.015$^{Bc}$ |
| Sucrose | 5% | Aerobiosis | 0.671±0.014$^{Ad}$ | 0.129±0.002$^{Aa}$ | 0.220±0.006$^{Ab}$ | 0.220±0.001$^{Ab}$ | 0.374±0.015$^{Ac}$ |
| | 10% | | 0.671±0.006$^{Ad}$ | 0.137±0.006$^{Aa}$ | 0.222±0.011$^{Ab}$ | 0.230±0.011$^{Bb}$ | 0.372±0.007$^{Ac}$ |
| | 20% | | 0.684±0.015$^{Ad}$ | 0.127±0.006$^{Aa}$ | 0.209±0.015$^{Ab}$ | 0.225±0.005$^{Bb}$ | 0.397±0.012$^{Bc}$ |
| | 5% | Anaerobiosis | 0.778±0.012$^{Bd}$ | 0.157±0.005$^{Ba}$ | 0.356±0.006$^{Bc}$ | 0.197±0.012$^{Ab}$ | 0.358±0.013$^{Ac}$ |
| | 10% | | 0.807±0.008$^{Cc}$ | 0.186±0.006$^{Ca}$ | 0.352±0.020$^{Bb}$ | 0.195±0.004$^{Aa}$ | 0.357±0.018$^{Ab}$ |
| | 20% | | 0.868±0.004$^{Dd}$ | 0.189±0.007$^{Ca}$ | 0.360±0.010$^{Bc}$ | 0.236±0.009$^{Bb}$ | 0.352±0.009$^{Ac}$ |

**Table S5**. Biofilm production (expressed as OD value at 580 nm) of *L. plantarum* strains in MRS (aerobiosis and anaerobiosis conditions) with glucose, fructose, sucrose after incubation at 37° C for 24 h. Results are reported as mean ± standard deviation (n=3). For every sugar, different lowercase letters (a-c), in each row, and different uppercase letters (A-E), in each column, indicate significant differences ($p < 0.05$).
